# Insulin, Testosterone, and Albumin in Term and Preterm Breast Milk, Donor Milk, and Infant Formula

**DOI:** 10.3390/nu15061476

**Published:** 2023-03-19

**Authors:** Réka A. Vass, Edward F. Bell, Robert D. Roghair, Gabriella Kiss, Simone Funke, Szilvia Bokor, Dénes Molnár, Attila Miseta, József Bódis, Kálmán Kovács, Tibor Ertl

**Affiliations:** 1 Department of Obstetrics and Gynecology, Medical School University of Pécs, 7624 Pécs, Hungary; 2 MTA-PTE Human Reproduction Scientific Research Group, University of Pécs, 7624 Pécs, Hungary; 3 Obstetrics and Gynecology, Magyar Imre Hospital Ajka, 8400 Ajka, Hungary; 4 Stead Family Department of Pediatrics, University of Iowa, Iowa City, IA 52242, USArobert-roghair@uiowa.edu (R.D.R.); 5 Department of Laboratory Medicine, Medical School University of Pécs, 7624 Pécs, Hungary; 6 Department of Pediatrics, Medical School University of Pécs, 7624 Pécs, Hungary

**Keywords:** holder pasteurization, insulin, testosterone, preterm milk, term milk, donor milk, infant formula, albumin/protein ratio

## Abstract

Background: Infants have three options for feeding: their own mother’s breast milk, donor milk, or infant formula. Insulin, testosterone, total protein, and albumin levels were measured in breast milk samples from the first 6 months of lactation, in donor milk samples, and in different infant formulas. Methods: Mothers who gave birth to term (*n* = 19) or preterm (*n* = 19) infants were recruited to collect breast milk samples during the first 6 months of lactation. The Breast Milk Collection Center (Unified Health Institution, Pécs, Hungary) provided 96 donor milk (DM) samples for analysis in our study. Insulin, testosterone, total protein, and albumin levels were measured in breast milk, donor milk, and infant formulas. Results: During the first 2 months of lactation, the concentration of insulin was lower (−27.4%) while the testosterone concentration was higher (+20.8%) compared to the period between the 3rd and 6th months only in the preterm breast milk samples. The infant formulas examined did not contain insulin or testosterone. Holder pasteurization (HoP) did not influence the level of testosterone in human milk, although HoP decreased the insulin (−53.6%) and albumin (−38.6%) concentrations. Conclusions: Diet impacts the hormone intake of infants, underlining the importance of breastfeeding and the possible supplementation of formula-fed infants.

## 1. Introduction

Breast milk provides a unique source of nutrition and biologically active agents such as hormones to the developing infant. Maternal hormones typically influence human life, from the beginning of intrauterine development through to transplacental circulation. Preterm birth disrupts intrauterine development. For these infants, breastfeeding is the only source of maternal hormones and other essential factors [1,2,3]. Infants have three feeding options: their own mother’s breast milk, donor milk (DM), or infant formula. Maternal milk has different compositions depending on whether the infant was born at term or preterm [4]. Other factors, such as body mass index (BMI), also influence the composition of breast milk. Donor milk, to ensure microbiological safety, is Holder pasteurized, and this procedure is known to influence the composition of DM [4,5].

Insulin, as an anabolic hormone, modulates blood glucose levels and protects against hypo- and hyperglycemic states. Infants born very prematurely have functionally and structurally immature gastrointestinal tracts [6]. Enteral insulin has local effects, including increasing intestinal maturation and ameliorating microbiome diversity [7,8]. Preterm infants rely on external factors, especially when breastfeeding is impeded in clinical practice [9]. In a recent study, preterm infants received low-dose recombinant human (rh) insulin (400 μIU/mL milk), high-dose rh insulin (2000 μIU/mL milk), or placebo for 4 weeks to investigate the use of rh insulin as a supplement. Very preterm infants receiving either dose of insulin achieved full enteral feedings more rapidly than infants receiving the placebo [10].

Testosterone is a key hormone of the male reproductive system and influences neurodevelopment [11]. Androgen level elevation occurs postnatally. This elevation, called minipuberty, is thought to contribute to neurobehavioral sexual differentiation [12]. In adults, orally administered testosterone can be absorbed, especially when given with fats or meals, but its bioavailability is considerably reduced by first-pass hepatic metabolism [13]. Testosterone promotes skeletal muscle hypertrophy by modulating the commitment of pluripotent mesenchymal cells [14].

Many of the known physiologic advantages of breast milk are provided by proteins. As a circulatory protein, albumin has various physiological functions, such as maintaining microvascular integrity, regulating metabolic processes and oncotic pressure, providing antioxidant activities, binding ligands to substances, and having anticoagulant effects [15].

When breastfeeding is not feasible, donor milk is the recommended form of infant feeding [1]. The composition of DM is modified by Holder pasteurization (HoP), but, previously, only one work had analyzed the effect of HoP on testosterone levels in human milk [16]. Holder pasteurization reduces the levels of insulin [16,17], thyroxine [18], cortisol, and cortisone in breast milk [19]. In the past few years, several research groups have investigated the effect of HoP on the concentrations of other substances in breast milk. Adiponectin and erythropoietin (EPO) concentrations have both been reported to be decreased significantly after Holder pasteurization, [5,17,19].

Three options are available for infant feeding: the mother’s own breast milk, donor milk, or infant formula. The examined hormones, insulin and testosterone, control numerous developmental checkpoints through their neuromodulator and metabolic roles. We aimed to analyze the presence of insulin, testosterone, total protein, and albumin in breast milk, donor milk, and infant formula samples.

## 2. Materials and Methods

Mothers were recruited after giving birth at the Department of Obstetrics and Gynecology, University of Pécs (Pécs, Hungary). Mothers of preterm (*n* = 19) and term (*n* = 19) infants collected monthly breast milk samples from the 4th postpartum week until the 6th postpartum month. Between 1 p.m. and 3 p.m. at home, the mothers pumped their entire breast expression into sterile polypropylene bottles. From the bottle, 5 mL was poured into a sterile polypropylene tube and stored until analysis. The Regional and Local Research Ethics Committee of the University of Pécs, Hungary (PTE KK 7072-2018) gave us permission to conduct our study.

To investigate the effect of HoP on breast milk composition, 96 registered donor mothers were recruited from the Breast Milk Collection Center of the Unified Health Institution at Pécs, Hungary. Mothers donated freshly pumped breast milk according to the center’s protocol. We collected breast milk samples on 10 random occasions. Samples were taken individually and then pooled and Holder pasteurized (30 min at 62.5 °C) at the Unified Health Institution (Pécs, Hungary). Three samples were taken from the Holder pasteurized donor milk pool and stored at −80 °C until further analysis. The samples were sonicated in order to disrupt the milk fat globule membranes [16,18] and then centrifuged at 15,000× *g* for 15 min. The skimmed milk was then transferred into polypropylene tubes for analysis according to the previously described preparation methods [4].

Three infant formulas were tested to analyze their insulin, testosterone, total protein, and albumin levels: Nutricia Milumil Pepti Pronutra (Danone, Paris, France), Beba Optipro Hypoallergenic (HA) Start (Nestlé, Vevey, Switzerland), and Beba Optipro HA Pre (Nestlé, Vevey, Switzerland). On 7 different days, samples were taken at the Neonatal Intensive Care Unit in the morning between 8 and 9 a.m. and stored in sterile polypropylene tubes at −80 °C.

For insulin measurement, 20 µL from the samples was mixed with a biotinylated monoclonal insulin-specific antibody and a monoclonal insulin-specific antibody labeled with a ruthenium complex to form a sandwich complex. After the addition of streptavidin-coated microparticles, the complex binds to the solid phase through the interaction of streptavidin and biotin. Then, the mixture is aspirated and the microparticles are magnetically captured to the surface of the electrode. The remnant was removed with ProCell. A photomultiplier measured the chemiluminescent emission. The results were calculated via a calibration curve generated by instrument-specific 2-point calibration and a master curve provided via the reagent barcode. The detection range in the case of insulin was 1.39–6945 pmol/L. The lower detection limit was 0.20 µU/mL. Parallel with our samples, quality controls were tested.

After assay calibration, the testosterone levels in the samples were detected using a 1:3 dilution protocol. When the results were above 35 nmol/L, the machine automatically added specimen diluent. The minimum sample volume was calculated by the system. After loading the samples, assay-specific diluent was added followed by washing steps. Pretrigger and trigger solutions were added and, to determine testosterone quantity, the chemiluminescent emission was measured. The results were given with the use of a 4-parameter logistic curve. The detection range in the case of testosterone was 1.15–64.57 nmol/L and the calibrator range was 0.01–15.0 ng/mL.

The ARCHITECT i system was applied for the measurements, following the manufacturer’s instructions. All measurements were performed using a fully automatized Cobas e 411 analyzer system (Roche Diagnostics, Rotkreuz, Switzerland).

The biuret method was used to measure the total protein levels in the breast milk samples. This colorimetric technique determines total protein levels by spectrophotometry at 540–560 nm [4]. An immunoturbidimetric assay was used to measure albumin (Roche Diagnostics, Rotkreuz, Switzerland). Anti-albumin antibodies reacted with the samples’ antigens and formed complexes. The samples were detected turbidimetrically in an absolutely automatized way, and quality controls were also applied.

For statistical analysis, we used GraphPad (La Jolla, CA, USA). Shapiro–Wilk tests were performed to test the normality of the data. Statgraphics Centurion XVII version 17.0.16 (Statpoint Technologies, Inc., Warrenton, VA, USA) software was used to perform the statistical data analyses. Data were analyzed by using t-tests or ANOVA. A repeated measures one-way ANOVA test (with a post hoc Dunnett test) was used to compare the effect of HoP on the concentration of the examined hormones. Differences were considered statistically significant when *p*-values were <0.05. The study was powered to detect moderate effect sizes (Cohen’s d = 0.6). The results are presented as mean ± SEM.

## 3. Results

### 3.1. Maternal Demographics

**Figure 1:**
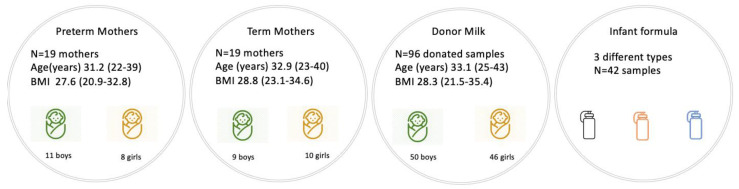
Measured samples and maternal data.

All mothers conceived spontaneously. Maternal demographics, e.g., maternal age, body mass index (BMI), infant sex, and mode of delivery, did not differ between the mothers who gave birth to term infants and those who gave birth to preterm infants. The average gestational age in the preterm group (33.5 ± 1.1 weeks) was significantly lower compared to the term group (39.7 ± 0.6 weeks). The mothers participating in the study were all of White race. None of the mothers had chronic health conditions, e.g., diabetes previously or throughout their pregnancy or lactation period, and none of them followed any special diet or took any medication during lactation. The mothers participating in our study were taking prenatal vitamins and magnesium, folic acid, or iron supplementation in recommended doses. Maternal BMI was measured in the 4th postpartum week when the first milk sample was collected from the participants. The time interval between childbirth and donation for the donor mothers was 125.4 ± 13.1 days (Figure 1).

### 3.2. Components in Breast Milk

The breast milk produced for the preterm infants had consistent total protein concentration and albumin levels during the first six months of lactation. The insulin concentration throughout the first 2 months of lactation was lower than in the period between the 3rd and 6th month. Throughout the first 2 months of breastfeeding, the testosterone levels in the breast milk were significantly higher compared to the values measured between the 3rd and 6th month (Table 1).

When comparing the average concentration values measured in the preterm and term breast milk samples, the total protein, insulin, and testosterone levels did not differ in the preterm and term breast milk during the first 6 months of lactation. We did not find monthly differences when comparing the concentration of total protein, insulin, and testosterone in the preterm and term samples from the same time period (data not shown). The albumin level in the preterm milk was significantly higher than in the term milk every month during the first 6 months of lactation. The albumin/total protein ratio was significantly lower in the term milk throughout the investigation period (Table 2). We found no effect of infant sex on milk insulin, testosterone, albumin, and total protein levels (data not shown).

Holder pasteurization is the most widely used technology to ensure the microbiological safety of donor milk. After this procedure, the insulin and albumin levels were significantly decreased, while the testosterone and total protein concentrations were unchanged (Table 3).

**Figure 2:**
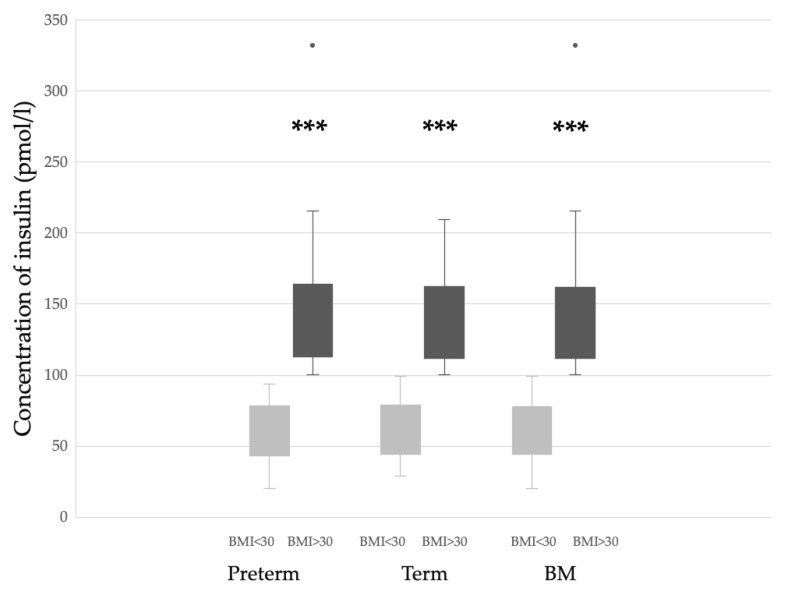
Impact of BMI on the insulin concentration of preterm or term breast milk and the combined group of breast milk (BM) samples. *** *p* < 0.001 versus BMI < 30.

It is known that BMI has an impact on insulin levels in breast milk [16]. This observation was confirmed by the present data. Regardless of gestational age, mothers with a BMI over 30 secreted significantly higher amounts of insulin into their breast milk (Figure 2). Testosterone concentration did not differ based on maternal BMI.

### 3.3. Infant Formula Analysis

The infant formulas currently in use at the Department of Neonatology, University of Pécs, are Milumil Pepti Pronutra, Beba Optipro HA Start, and Beba Optipro HA Pre. Insulin and testosterone were not detected above the lower limits of detection in the samples (*n* = 21). The formulas had an average of 10.39 ± 4.58 g/L total protein and 302.51 ± 66.87 mg/L albumin levels which did not differ significantly from the term and preterm breast milk.

## 4. Discussion

We have provided previously unavailable data on the presence of testosterone in preterm and term breast milk during the first 6 months of lactation. Insulin levels were significantly lower, while testosterone concentrations were significantly higher in the first 2 months of breastfeeding compared to the second part of the examination period (3rd–6th month) in the breast milk produced for preterm infants. Preterm and term breast milk have similar insulin and testosterone concentrations throughout the first 6 months of lactation. We confirmed previous data showing that Holder pasteurization decreases the levels of insulin and albumin in donor milk [16,18]. HoP does not influence testosterone and total protein concentrations. Infant formulas do not contain insulin or testosterone.

Insulin is known to have multifunctional regulatory effects, controlling hypo- and hyperglycemic states [20], and to participate in neurodevelopment, short- and long-term memory unification, and strengthening in the hippocampus [21]. The level of insulin as a peptide hormone, not surprisingly, was decreased (−53.6%) after HoP. Analyzing a similar donor-milk-bank population, Ley and coworkers also found a significant decrease in insulin (−46% decrease), whereas in our previous work, we detected a 13% reduction [22]. Presumably, this difference was caused by the detection method. Ley et al. and the present work applied a chemiluminescence array, while our previous work used a microbead array [16]. Maternal obesity influences the composition of breast milk [23], and the present results confirm the previous observation that the breast milk of obese mothers contains a higher insulin concentration, raising the question of whether this may contribute to neonatal hypoglycemia [20,24]. Infant growth rate did not differ based on maternal BMI in our study groups. Our data strengthen the finding that maternal BMI is independently associated with insulin levels since our study excluded women with diabetes before or during pregnancy. The transportation of insulin into human milk is an active process and it is protected from degradation. Presumably, insulin plays a functional and developmental role in the infant. Mosinger et al. reported that intact insulin was able to retain biological activity when ingested, cross from human milk into the bloodstream, and decrease blood glucose levels [25]. Enteral insulin promotes intestinal maturation. Recombinant human insulin may be considered for enteral administration as a supplement to breast milk, donor milk, and formula milk. A multicenter, double-blind, placebo-controlled randomized clinical trial was performed at 46 neonatal intensive care units in Europe, Israel, and the U.S. Premature infants born between 26 and 32 weeks of gestation were enrolled in the study. The results of this clinical investigation revealed that enteral administration of two different rh insulin doses was safe. Time of full enteral feeding was significantly reduced compared with the placebo in preterm infants with a GA of 26 to 32 weeks [10]. In agreement with animal experiments, Mank et al. investigated intestinal lactase activity, which was found to be significantly higher in preterm infants receiving enteral rh insulin [7,8,9]. Insulin receptors were detected on both the apical and basolateral enterocyte membranes of different animals and these receptors possibly control the effect of enteral insulin in the intestine [26,27,28,29,30]. Limited information is available about insulin receptor development on enterocyte membranes in fetuses [31]. These findings and previous investigations [32,33] support the supplementation of human milk and preterm formula with insulin.

Testosterone has a known neuro- and immunomodulatory role. Moreover, its elevation in plasma samples has been described in male infants during minipuberty, which was associated with sex-typed behavior at 14 months [34,35]. Testosterone increases hemoglobin and hematocrit levels, which is associated with the stimulation of erythropoietin (EPO) and decreased ferritin and hepcidin concentrations. The authors concluded that testosterone enhances erythropoiesis by stimulating EPO. They observed that testosterone increased iron utilization for erythropoiesis [36]. During fetal development, the EPO receptor is widely distributed, and EPO is considered a neuroprotective agent in hypoxia [37]. In early life, hepatic immaturity provides the possibility for steroid hormones to reach the systemic circulation in relatively low concentrations. In adults, oral testosterone is able to be absorbed, but the bioavailability may be reduced by the first-pass effect of hepatic metabolism [38]. Our present work strengthens the previous observation of our research group that testosterone is present in breast milk [16], but the novel aspect of our current study is that we followed mothers for 6 months, the time of recommended exclusive breastfeeding, and observed that testosterone is present in equal amounts in the breast milk of mothers of term and preterm infants. This confirmed the previous results of our research group in that testosterone concentrations are similar in preterm and term breast milk. We also demonstrated that three infant formulas did not contain testosterone.

Gastric and intestinal digestion of protein and other components are attenuated during the first few postnatal months. In the newborn infant’s gastrointestinal tract, the cell–cell connections and barrier functions are immature [39,40], presumably promoting the absorption or elongated presence of breast milk components in the gastrointestinal tract. Hormones have multifactorial regulatory effects such as nutritional and maturational signals which control differentiation and tissue development through the gut–brain axis [41,42]. 

If the mother’s own milk is not available in the neonatal intensive care unit, donor milk is the recommended choice for preterm infants [1]. The use of donor milk reduces neonatal diseases, such as necrotizing enterocolitis, compared to formula milk [43]. Before feeding it to infants, donor milk is submitted to HoP in human milk banks to ensure microbiological safety. The impact of HoP on the concentration of various hormones differs [4,5,16,44]. Although the procedure reduces the concentration of components, such as insulin and albumin in donor milk, some factors keep their concentration after the procedure. Compared to infant formula, donor milk provides a variety of essential hormones to ensure development and promote ideal postnatal development [45].

Its elevated presence in preterm milk suggests that it may satisfy an elevated functional need developed because of prematurity. Albumin acts as a plasma carrier by non-specifically binding several hydrophobic steroid hormones; it also transports thyroid hormones and fatty acids. With the present results, we strengthen the previous findings about the presence of albumin in human breast milk, providing a balanced complement of essential amino acids to the infant [46,47].

One of our study’s limitations is that the milk samples were sonicated to disrupt milk fat globules, which allows proteins to access the aqueous phase. Thus, the removal of the fat layer may have caused the underestimation of hormone levels.

## 5. Conclusions

Different feeding options provide altered nutrition and hormonal intake for infants. Prematurity causes the interruption of continuous hormonal exposure during intrauterine development. Our results have shown that testosterone is present in similar amounts in human milk during the first 6 months of lactation in preterm and term breast milk, and its concentration is not influenced by Holder pasteurization. Our results confirm earlier findings in that obese mothers secrete insulin at significantly higher concentrations into their milk, regardless of gestational age. These data on the hormonal content of preterm and term breast milk, donor milk, and infant formula call attention to the potential for supplementation of hormones and other biologically active factors during postnatal life.

## Figures and Tables

**Table 1:** Total protein, insulin, testosterone, and albumin in preterm (*n* = 114) breast milk samples.

	1st–2nd Month	3rd–6th Month	*p* Value
Total protein g/L	10.5 ± 0.6	9.9 ± 0.7	0.2309
Insulin pmol/L	86.4 ± 12.2	119.1 ± 12.7	0.0439
Testosterone pmol/L	63.3 ± 5.2	50.1 ± 4.6	0.0212
Albumin mg/L	361.4 ± 22.8	351.7 ± 11.9	0.2613

**Table 2:** Average concentration of total protein, insulin, testosterone, and albumin in preterm (*n* = 114) and term (*n* = 114) breast milk samples during the first 6 months of lactation.

	Preterm	Term	*p* Value
Total protein g/L	10.29 ± 0.57	12.02 ± 1.03	0.2813
Insulin pmol/L	109.1 ± 9.8	96.7 ± 5.9	0.2617
Testosterone pmol/L	54.3 ± 3.9	60.1 ± 5.2	0.3901
Albumin mg/L	349.4 ± 21.3	258.9 ± 11.6	0.0032
Albumin/protein ratio (g/L)	0.032 ± 0.001	0.021 ± 0.011	0.0011

**Table 3:** Impact of Holder pasteurization on the concentrations of insulin and testosterone in donor milk (*n* = 96).

	Raw	HoP	*p* Value
Total protein g/L	9.5 ± 0.2	9.8 ± 0.1	0.1091
Insulin pmol/L	97.4 ± 13.7	45.1 ± 3.1	0.0002
Testosterone umol/L	43.1 ± 4.3	42.4 ± 0.9	0.0892
Albumin mg/L	293.7 ± 12.4	211.9 ± 10.2	0.0026

## Data Availability

Data applied in this study are available upon request from the corresponding author.
